# Supplementary figures and images for: Cross-comparative analysis of evacuation behavior after earthquakes using mobile phone data
Source: PLoS One. 2019 Feb 20;14(2):e0211375. doi: 10.1371/journal.pone.0211375 (PMC6382263; doi:10.1371/journal.pone.0211375)

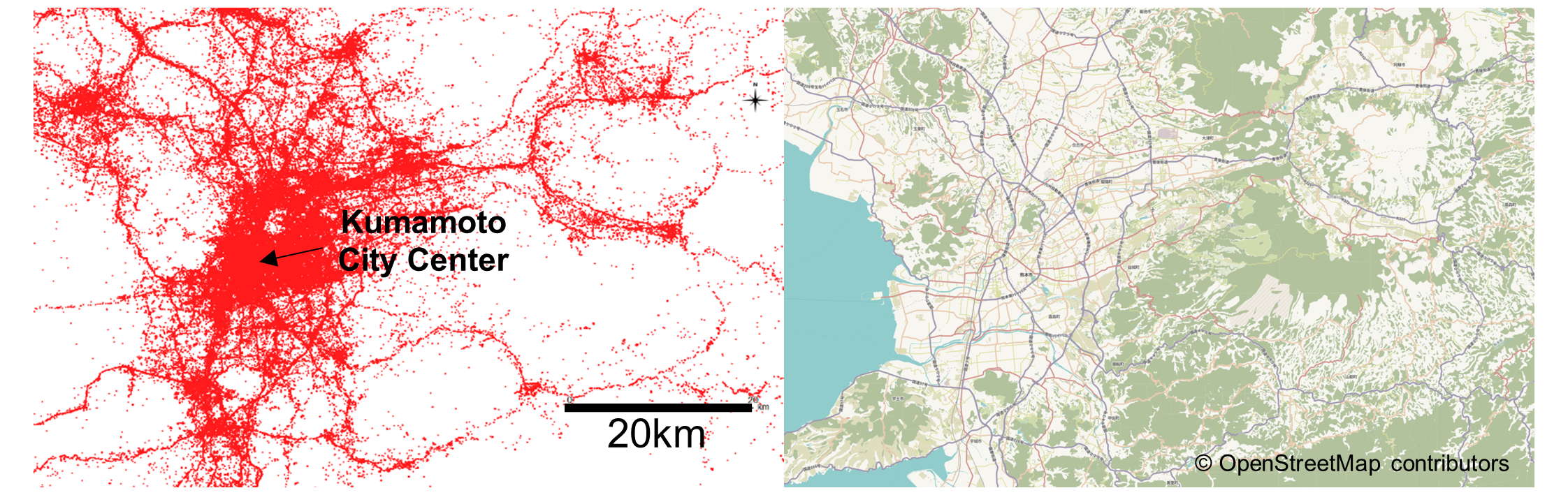

Supplement: S1 Fig — Left: GPS data obtained during 1 day plotted onto a white map in Kumamoto area. Right: OpenStreetMap data of Kumamoto area, showing the road networks [46]. The mobile phone data is dense in both spatial and temporal aspects to analyze the detailed mobility of individuals. (PNG) [file pone.0211375.s001.png]

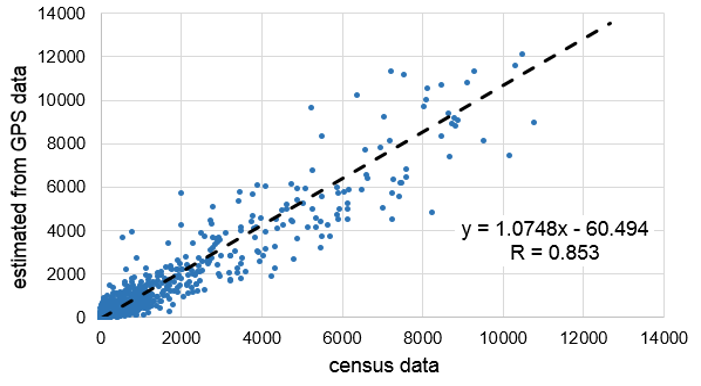

Supplement: S2 Fig — Estimated population and the population obtained from the census. The blue dots correspond to one grid mesh (1000m size) respectively. (PNG) [file pone.0211375.s002.png]

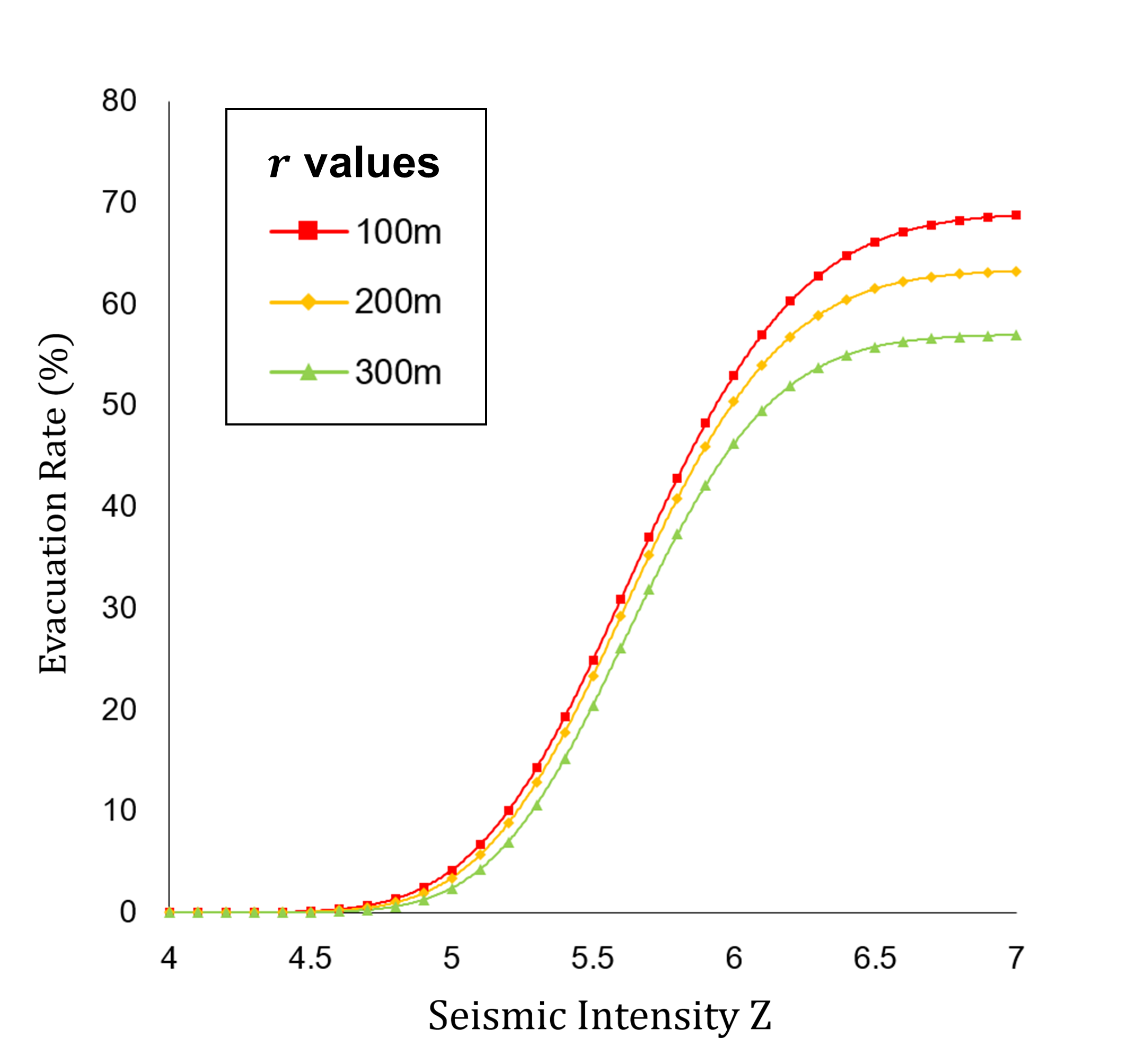

Supplement: S3 Fig — Fragility curves with different r parameter values. Although the estimated parameters vary under different parameters, the general findings are not affected, where the fragility curves fit the result well. (TIF) [file pone.0211375.s003.tif]

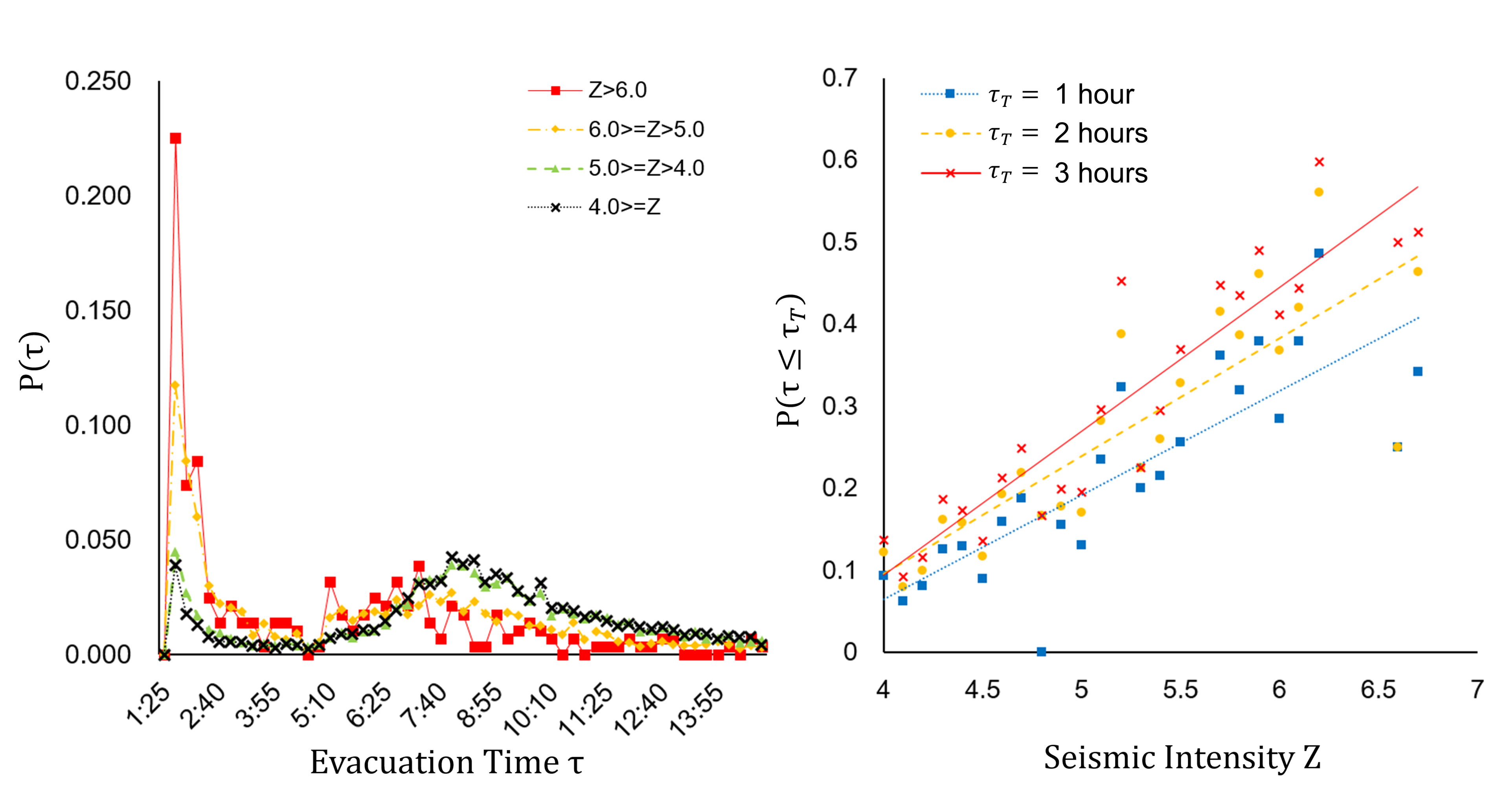

Supplement: S4 Fig — Evacuation timing of individuals after the Kumamoto earthquake. Results show that the higher the seismic intensity, individuals evacuate more quickly. (TIF) [file pone.0211375.s004.tif]
